# Poly[[tetra­kis­(μ_2_-pyrazine *N*,*N*′-dioxide-κ^2^ *O*:*O*′)neodymium(III)] tris­(perchlorate)]

**DOI:** 10.1107/S1600536810031818

**Published:** 2010-08-18

**Authors:** Benjamin G. Quinn-Elmore, James D. Buchner, Keith B. Beach, Jacqueline M. Knaust

**Affiliations:** a Allegheny College, 520 North Main St., Meadville, PA 16335, USA

## Abstract

The title three-dimensional coordination network, {[Nd(C_4_H_4_N_2_O_2_)_4_](ClO_4_)_3_}_*n*_, is isostructural to that of other lanthanides. The Nd^+3 ^cation lies on a fourfold roto-inversion axis. It is coordinated in a distorted square-anti­prismatic fashion by eight O atoms from bridging pyrazine *N*,*N*′-dioxide ligands. There are two unique pyrazine *N*,*N*′-dioxide ligands. One ring is located around an inversion center, and there is a twofold rotation axis at the center of the other ring. There are also two unique perchlorate anions. One is centered on a twofold rotation axis and the other on a fourfold roto-inversion axis. The perchlorate anions are located in channels that run perpendicular to (001) and (110) and inter­act with the coordination network through C—H⋯O hydrogen bonds.

## Related literature

For the isostructural La, Ce, Pr, Sm, Eu, Gd, Tb and Y coord­ination networks, see: Sun *et al.* (2004 ▶). For the isostructural Dy, Ho, Er coordination networks, see: Quinn-Elmore *et al.* (2010 ▶); Buchner *et al.* (2010**a* ▶,b* ▶), respectively. For a lanthanum 4,4′-bipyridine *N,N*′-dioxide coordination network of similar topology, see: Long *et al.* (2001 ▶). For additional discussions on *Ln* ^3+^ (*Ln* = lanthanide) coordination networks with aromatic *N,N*′-dioxide ligands, see: Cardoso *et al.* (2001 ▶); Hill *et al.* (2005 ▶). For background information on the applications of coordination networks, see: Roswell & Yaghi (2004 ▶); Rosi *et al.* (2003 ▶); Seo *et al.* (2000 ▶).
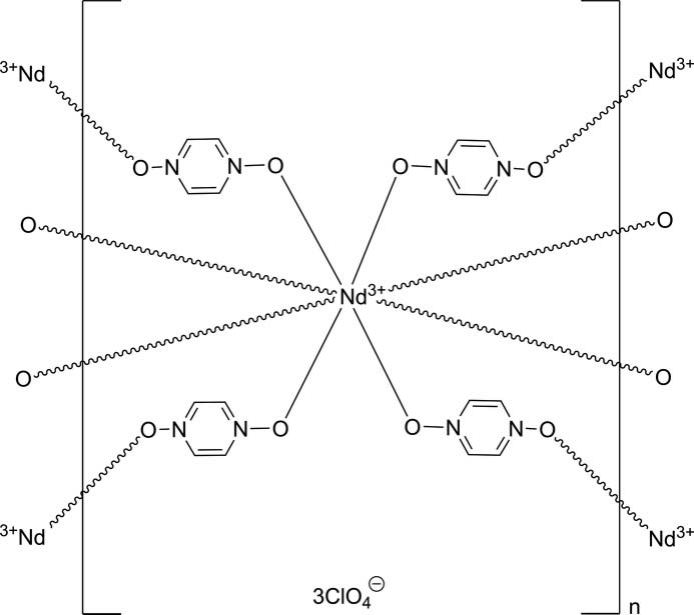


## Experimental

### 

#### Crystal data

[Nd(C_4_H_4_N_2_O_2_)_4_](ClO_4_)_3_
*M* *_r_* = 890.96
Tetragonal, 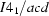

*a* = 15.3804 (4) Å
*c* = 22.9843 (12) Å
*V* = 5437.1 (3) Å^3^
*Z* = 8
Mo *K*α radiation
μ = 2.32 mm^−1^
*T* = 100 K
0.23 × 0.23 × 0.18 mm

#### Data collection

Bruker SMART APEX CCD diffractometer
Absorption correction: multi-scan (*SADABS*; Bruker, 2001 ▶) *T*_min_ = 0.593, *T*_max_ = 0.659
30711 measured reflections
2086 independent reflections
1842 reflections with *I* > 2σ(*I*)
*R*_int_ = 0.024

#### Refinement

*R*[*F* ^2^ > 2σ(*F* ^2^)] = 0.038
*wR*(*F* ^2^) = 0.106
*S* = 1.10
2086 reflections
110 parameters
H-atom parameters constrained
Δρ_max_ = 2.27 e Å^−3^
Δρ_min_ = −1.95 e Å^−3^

### 

Data collection: *SMART* (Bruker, 2007 ▶); cell refinement: *SAINT-Plus* (Bruker, 2007 ▶); data reduction: *SAINT-Plus*; program(s) used to solve structure: *SHELXS97* (Sheldrick, 2008 ▶); program(s) used to refine structure: *SHELXL97* (Sheldrick, 2008 ▶); molecular graphics: *X-SEED* (Barbour, 2001 ▶); software used to prepare material for publication: *X-SEED*.

## Supplementary Material

Crystal structure: contains datablocks I, global. DOI: 10.1107/S1600536810031818/zl2298sup1.cif

Structure factors: contains datablocks I. DOI: 10.1107/S1600536810031818/zl2298Isup2.hkl

Additional supplementary materials:  crystallographic information; 3D view; checkCIF report

## Figures and Tables

**Table 1:** Hydrogen-bond geometry (Å, °)

*D*—H⋯*A*	*D*—H	H⋯*A*	*D*⋯*A*	*D*—H⋯*A*
C2—H2⋯O2^i^	0.95	2.55	3.326 (3)	139
C2—H2⋯O5	0.95	2.43	3.194 (7)	137
C3—H3⋯O1	0.95	2.59	3.331 (3)	135
C3—H3⋯O3	0.95	2.51	3.260 (3)	136
C4—H4⋯O3^ii^	0.95	2.41	3.289 (3)	154

Symmetry codes: (i) 
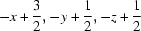
; (ii) 
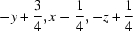
.

**Fig. 1.**
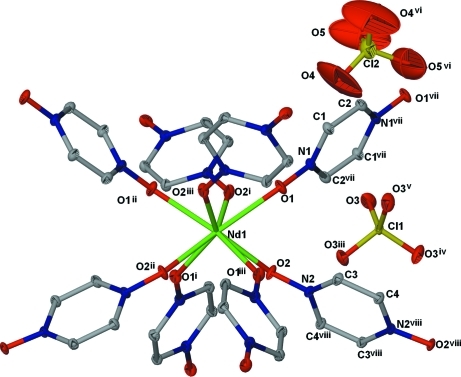
The coordination environment of the Nd+3 cation in title compound with atom labels and 50% probability displacement ellipsoids. Hydrogen atoms have been omitted for clarity. Symmetry codes: (i) y+1/4, x-1/4, -z+3/4; (ii) -y+3/4, -x+3/4, -z+3/4; (iii) -x+1, -y+1/2, z; (iv) -y+3/4, x-1/4, -z+1/4; (v) y+1/4, -x+3/4, -z+1/4; (vi) y+3/4, x-3/4, -z+1/4; (vii) -x+3/2, -y+1/2, -z+1/2; (viii) x, -y+1, -z+1/2.

**Fig. 2.**
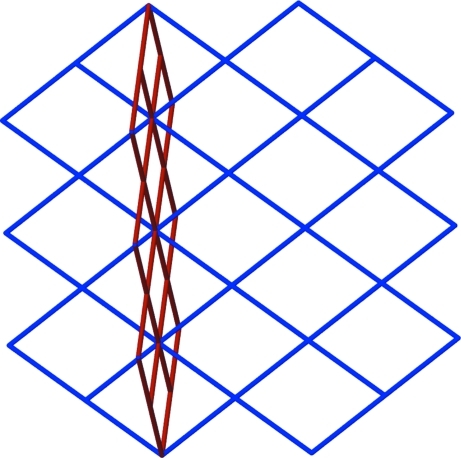
Schematic representation of the network topology seen in {[Nd(C4H4N2O2)4](ClO4)3}n. The net shown in red is parallel to the (1 0 0) plane, and the net shown in blue is parallel to the (0 1 0) plane.

**Fig. 3.**
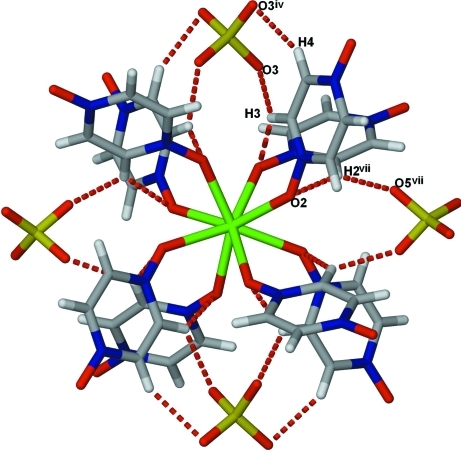
C—H···O hydrogen bonding interactions between pyrazine N,N'-dioxide ligands and between perchlorate anions and pyrazine N,N'-dioxide ligands. Hydrogen bonds are shown as dashed lines. Symmetry codes: (iv) -y+3/4, x-1/4, -z+1/4;; (vi) y+3/4, x-3/4, -z+1/4; (vii) -x+3/2, -y+1/2, -z+1/2 Color scheme: Nd: green, C: grey, H: white, N:blue, O: red, Cl: yellow.

**Fig. 4.**
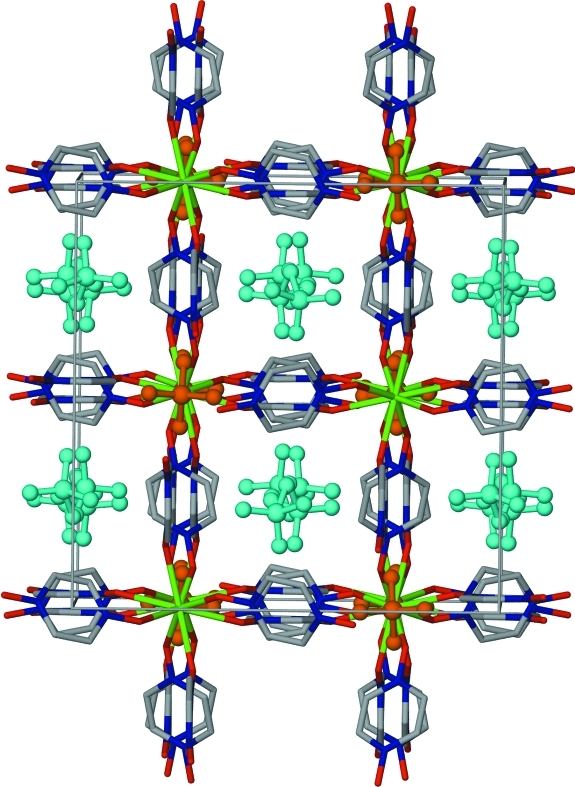
Packing of the title compound viewed perpendicular to the (0 0 1) with anions shown in ball and stick representation. In these channels there are only anions containing Cl2. Hydrogen atoms have been omitted for clarity. Color scheme: Nd: green, C: grey, H: white, N:blue, O: red, anions containing Cl1: orange, and anions containing Cl2: aqua.

**Fig. 5.**
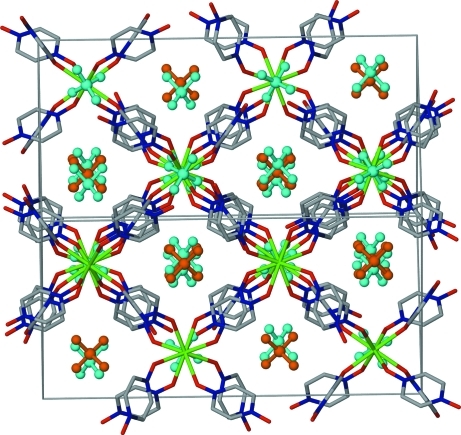
Packing of the title compound viewed perpendicular to the (1 1 0) plane with anions shown in ball and stick representation. The the perchlorate anions in these channels alternate between those containing Cl1 and Cl2. Hydrogen atoms have been omitted for clarity. Color scheme: Nd: green, C: grey, H: white, N:blue, O: red, anions containing Cl1: orange, and anions containing Cl2: aqua.

## supplementary crystallographic information

### Comment

The synthesis of lanthanide coordination networks has been of recent interest due to the potential of the flexible coordination sphere of the Ln^+3^ metal ions to produce coordination networks with new, unusual, or high connectivity topologies (Hill *et al.* 2005, Long *et al.* 2001, and Sun *et al.* 2004). Coordination networks with both a high connectivity topology and an open framework have potential for applications in areas such as absorption, ion exchange, or catalysis (Roswell *et al.* 2004, Rosi *et al.* 2003, and Seo *et al.* 2000). Aromatic *N,N*'-dioxide ligands have been attractive candidates for use with Ln^+3^ cations as the O-donor atoms of the ligand are complementary to the hard acid character of the lanthanide cations (Cardoso *et al.* 2001, Hill *et al.* 2005, Long *et al.* 2001, and Sun *et al.* 2004).

The description of the structure of the title compound is part of a series of consecutive papers on three-dimensional coordination networks of the type {[Ln(C_4_H_4_N_2_O_2_)_4_](ClO_4_)_3_}_n_, with Ln = Nd (this publication), Dy (Quinn-Elmore *et al.* 2010), Ho (Buchner *et al.* 2010*a*) and Er (Buchner *et al.* 2010*b*), respectively. All four compounds are also isostructural to the previously reported La, Ce, Pr, Sm, Eu, Gd, Tb and Y coordination networks (Sun *et al.* 2004).

The asymmetric unit of the title compound contains one quarter of a Nd^+3^ cation, half of two coordinated pyrazine *N,N*'-dioxide ligands, a quarter of one perchlorate anion, and a half of another perchlorate anion (Figure 1). The Nd^+3 ^cation lies on a fourfold roto-inversion axis. One ligand (O1, N1, C1, C2) is located around an inversion center, and there is a twofold rotation axis at the center of the other (O2, N2, C3, C4). Both chlorine atoms of the perchlorate anions lie on special positions. Cl1 lies on a fourfold roto-inversion axis, and Cl2 is located on a twofold rotation axis. The high atomic displacement parameters for O4 and O5 bonded to Cl2, and the residual electron density around Cl2 indicate that this perchlorate anion is disordered; however, the disorder does not appear discreet. Only O4 and O5 are easily found in positions that agree with the site symmetry of the anion, therefore only one position was modeled.

The Nd^+3 ^cation is coordinated in a distorted square anti-prismatic fashion by eight O atoms from bridging pyrazine *N,N*'-dioxide ligands forming a three-dimensional coordination network. The network topology is similar to that which is seen in {[La(4,4'-bipyridine *N,N*'-dioxide)_4_](CF_3_SO_3_)_3_^.^4.2CH_3_OH}_n_ in that in can be considered as being composed of two sets of intersecting (4,4) nets (Long *et al.* 2001). The nets are perpendicular to one another, but they are canted. One set of nets lies parallel to the (1 0 0) plane, and the other set lies parallel to the (0 1 0) plane (Figure 2).

The title compound forms five unique C—H···O hydrogen bonds (Figure 3). There are two unique hydrogen bonds between pyrazine *N*,*N'*-dioxide ligands and another three hydrogen bonds between the perchlorate anions and pyrazine *N,N*'-dioxide ligands. The non-disordered perchlorate anion (Cl1 and O3) forms two unique hydrogen bonds with pyrazine *N,N*'-dioxide ligands resulting in a total of eight hydrogen bonds per ion with the network, but the disordered perchlorate (Cl2, O4, and O5) forms only one unique hydrogen bond with pyrazine *N,N*'-dioxide ligands resulting in a total of only two hydrogen bonds per ion with the network. As seen in the packing diagrams, the perchlorate anions are located in two sets of channels (Figures 4 and 5). In channels that run perpendicular to the (0 0 1) plane only anions containing Cl2 are present. (Figure 4), but in the channels that run perpendiclar to the (1 1 0) plane the anions containing Cl1 and Cl2 alternate (Figure 5).

### Experimental

Pyrazine *N,N*'-dioxide (0.025 g, 0.223 mmol) was dissolved in deionized water (1.5 ml) and methanol (1.5 ml). An aqueous solution of Nd(ClO_4_)_3_ (0.240 ml of a 0.1167 *M* solution, 0.028 mmol) was diluted with methanol (0.760 ml) and CH_2_Cl_2_ (2.5 ml). The pyrazine *N*,*N*'-dioxide solution was layered over the Nd(ClO_4_)_3_ solution, and the two solutions were allowed to slowly mix. Rose colored block-like crystals formed upon the slow evaporation of the resultant solution.

### Refinement

All H atoms were positioned geometrically and refined using a riding model with C—H = 0.95 Å and with *U_iso_*(H) = 1.2 times *U*_eq_(C).

### Crystal data

**Table:**

[Nd(C_4_H_4_N_2_O_2_)_4_](ClO_4_)_3_	*D*_x_ = 2.177 Mg m^−^^3^
*M**_r_* = 890.96	Mo *K*α radiation, λ = 0.71073 Å
Tetragonal, *I*4_1_/*a**c**d*	Cell parameters from 15053 reflections
Hall symbol: -I 4bd 2c	θ = 2.6–30.5°
*a* = 15.3804 (4) Å	µ = 2.32 mm^−^^1^
*c* = 22.9843 (12) Å	*T* = 100 K
*V* = 5437.1 (3) Å^3^	Block, rose
*Z* = 8	0.23 × 0.23 × 0.18 mm
*F*(000) = 3512	

### Data collection

**Table:**

Bruker SMART APEX CCD diffractometer	2086 independent reflections
Radiation source: fine-focus sealed tube	1842 reflections with *I* > 2σ(*I*)
graphite	*R*_int_ = 0.024
ω scans	θ_max_ = 30.5°, θ_min_ = 2.6°
Absorption correction: multi-scan (*SADABS*; Bruker, 2001)	*h* = −21→21
*T*_min_ = 0.593, *T*_max_ = 0.659	*k* = −21→21
30711 measured reflections	*l* = −32→32

### Refinement

**Table:**

Refinement on *F*^2^	Primary atom site location: structure-invariant direct methods
Least-squares matrix: full	Secondary atom site location: difference Fourier map
*R*[*F*^2^ > 2σ(*F*^2^)] = 0.038	Hydrogen site location: inferred from neighbouring sites
*wR*(*F*^2^) = 0.106	H-atom parameters constrained
*S* = 1.10	*w* = 1/[σ^2^(*F*_o_^2^) + (0.0535*P*)^2^ + 38.356*P*] where *P* = (*F*_o_^2^ + 2*F*_c_^2^)/3
2086 reflections	(Δ/σ)_max_ < 0.001
110 parameters	Δρ_max_ = 2.27 e Å^−^^3^
0 restraints	Δρ_min_ = −1.95 e Å^−^^3^

### Special details

**Table:**

Geometry. All esds (except the esd in the dihedral angle between two l.s. planes) are estimated using the full covariance matrix. The cell esds are taken into account individually in the estimation of esds in distances, angles and torsion angles; correlations between esds in cell parameters are only used when they are defined by crystal symmetry. An approximate (isotropic) treatment of cell esds is used for estimating esds involving l.s. planes.
Refinement. Refinement of *F*^2^ against ALL reflections. The weighted *R*-factor *wR* and goodness of fit *S* are based on *F*^2^, conventional *R*-factors *R* are based on *F*, with *F* set to zero for negative *F*^2^. The threshold expression of *F*^2^ > σ(*F*^2^) is used only for calculating *R*-factors(gt) *etc*. and is not relevant to the choice of reflections for refinement. *R*-factors based on *F*^2^ are statistically about twice as large as those based on *F*, and *R*- factors based on ALL data will be even larger.

### Fractional atomic coordinates and isotropic or equivalent isotropic displacement parameters (Å^2^)

**Table:**

	*x*	*y*	*z*	*U*_iso_*/*U*_eq_	
Nd1	0.5000	0.2500	0.3750	0.00589 (11)	
Cl1	0.5000	0.2500	0.1250	0.0119 (3)	
Cl2	0.72544 (6)	−0.02456 (6)	0.1250	0.0319 (3)	
O1	0.59191 (12)	0.21965 (14)	0.29303 (8)	0.0171 (4)	
O2	0.53338 (14)	0.39686 (12)	0.34324 (8)	0.0168 (4)	
O3	0.57573 (16)	0.24613 (16)	0.16135 (10)	0.0277 (5)	
O4	0.6477 (5)	−0.0172 (6)	0.1497 (5)	0.191 (4)	
O5	0.7895 (5)	−0.0023 (6)	0.1623 (5)	0.180 (5)	
N1	0.66963 (15)	0.23449 (15)	0.27293 (10)	0.0141 (4)	
N2	0.52833 (15)	0.44642 (14)	0.29745 (9)	0.0129 (4)	
C1	0.70832 (17)	0.17377 (17)	0.23897 (11)	0.0154 (5)	
H1	0.6797	0.1202	0.2315	0.018*	
C2	0.78886 (16)	0.18959 (17)	0.21540 (11)	0.0152 (5)	
H2	0.8155	0.1475	0.1910	0.018*	
C3	0.52715 (18)	0.41238 (16)	0.24314 (11)	0.0148 (5)	
H3	0.5264	0.3511	0.2380	0.018*	
C4	0.52702 (18)	0.46617 (16)	0.19547 (11)	0.0147 (5)	
H4	0.5260	0.4420	0.1574	0.018*	

### Atomic displacement parameters (Å^2^)

**Table:**

	*U*^11^	*U*^22^	*U*^33^	*U*^12^	*U*^13^	*U*^23^
Nd1	0.00627 (13)	0.00627 (13)	0.00511 (16)	−0.00034 (7)	0.000	0.000
Cl1	0.0146 (4)	0.0146 (4)	0.0065 (6)	0.000	0.000	0.000
Cl2	0.0304 (4)	0.0304 (4)	0.0349 (6)	−0.0118 (5)	0.0035 (3)	−0.0035 (3)
O1	0.0112 (8)	0.0256 (10)	0.0146 (8)	−0.0032 (7)	0.0052 (7)	−0.0036 (7)
O2	0.0283 (10)	0.0114 (8)	0.0107 (8)	−0.0031 (7)	−0.0038 (7)	0.0049 (6)
O3	0.0195 (11)	0.0479 (15)	0.0156 (10)	0.0057 (9)	−0.0054 (8)	−0.0033 (8)
O4	0.085 (5)	0.154 (7)	0.335 (12)	−0.015 (4)	0.138 (7)	−0.028 (7)
O5	0.091 (5)	0.130 (6)	0.317 (15)	0.007 (4)	−0.066 (7)	−0.135 (8)
N1	0.0116 (10)	0.0198 (10)	0.0110 (9)	−0.0011 (8)	0.0022 (8)	−0.0013 (8)
N2	0.0160 (10)	0.0117 (9)	0.0111 (9)	0.0000 (8)	−0.0017 (7)	0.0028 (7)
C1	0.0149 (11)	0.0172 (11)	0.0140 (11)	−0.0008 (9)	0.0023 (8)	−0.0029 (9)
C2	0.0135 (11)	0.0188 (12)	0.0132 (10)	0.0000 (9)	0.0013 (8)	−0.0027 (9)
C3	0.0206 (12)	0.0104 (10)	0.0134 (11)	−0.0018 (9)	−0.0019 (9)	0.0006 (8)
C4	0.0209 (12)	0.0114 (10)	0.0118 (10)	−0.0002 (9)	−0.0002 (9)	0.0006 (8)

### Geometric parameters (Å, °)

**Table:**

Nd1—O1^i^	2.4012 (18)	Cl2—O5^vi^	1.350 (7)
Nd1—O1^ii^	2.4012 (18)	O1—N1	1.302 (3)
Nd1—O1	2.4012 (18)	O2—N2	1.302 (3)
Nd1—O1^iii^	2.4012 (18)	N1—C1	1.355 (3)
Nd1—O2^i^	2.4286 (18)	N1—C2^vii^	1.358 (3)
Nd1—O2^ii^	2.4286 (18)	N2—C3	1.354 (3)
Nd1—O2	2.4286 (18)	N2—C4^viii^	1.354 (3)
Nd1—O2^iii^	2.4287 (18)	C1—C2	1.374 (3)
Cl1—O3	1.435 (2)	C1—H1	0.9500
Cl1—O3^iv^	1.435 (2)	C2—N1^vii^	1.358 (3)
Cl1—O3^iii^	1.435 (2)	C2—H2	0.9500
Cl1—O3^v^	1.435 (2)	C3—C4	1.373 (3)
Cl2—O4^vi^	1.328 (5)	C3—H3	0.9500
Cl2—O4	1.328 (5)	C4—N2^viii^	1.354 (3)
Cl2—O5	1.350 (7)	C4—H4	0.9500
			
O1^i^—Nd1—O1^ii^	76.62 (10)	O3^iv^—Cl1—O3^iii^	109.83 (10)
O1^i^—Nd1—O1	147.62 (10)	O3—Cl1—O3^v^	109.83 (10)
O1^ii^—Nd1—O1	112.75 (10)	O3^iv^—Cl1—O3^v^	108.76 (19)
O1^i^—Nd1—O1^iii^	112.75 (10)	O3^iii^—Cl1—O3^v^	109.83 (10)
O1^ii^—Nd1—O1^iii^	147.62 (10)	O4^vi^—Cl2—O4	109.6 (8)
O1—Nd1—O1^iii^	76.63 (10)	O4^vi^—Cl2—O5	115.9 (6)
O1^i^—Nd1—O2^i^	79.66 (7)	O4—Cl2—O5	111.4 (7)
O1^ii^—Nd1—O2^i^	73.01 (7)	O4^vi^—Cl2—O5^vi^	111.4 (7)
O1—Nd1—O2^i^	74.30 (6)	O4—Cl2—O5^vi^	115.9 (6)
O1^iii^—Nd1—O2^i^	137.92 (6)	O5—Cl2—O5^vi^	91.8 (11)
O1^i^—Nd1—O2^ii^	73.01 (7)	N1—O1—Nd1	141.71 (16)
O1^ii^—Nd1—O2^ii^	79.66 (7)	N2—O2—Nd1	140.80 (15)
O1—Nd1—O2^ii^	137.92 (6)	O1—N1—C1	119.1 (2)
O1^iii^—Nd1—O2^ii^	74.30 (6)	O1—N1—C2^vii^	120.8 (2)
O2^i^—Nd1—O2^ii^	145.02 (9)	C1—N1—C2^vii^	120.0 (2)
O1^i^—Nd1—O2	74.30 (6)	O2—N2—C3	121.3 (2)
O1^ii^—Nd1—O2	137.92 (6)	O2—N2—C4^viii^	119.0 (2)
O1—Nd1—O2	79.66 (7)	C3—N2—C4^viii^	119.7 (2)
O1^iii^—Nd1—O2	73.01 (7)	N1—C1—C2	120.1 (2)
O2^i^—Nd1—O2	72.37 (10)	N1—C1—H1	120.0
O2^ii^—Nd1—O2	118.92 (10)	C2—C1—H1	120.0
O1^i^—Nd1—O2^iii^	137.92 (6)	N1^vii^—C2—C1	119.9 (2)
O1^ii^—Nd1—O2^iii^	74.30 (6)	N1^vii^—C2—H2	120.1
O1—Nd1—O2^iii^	73.01 (7)	C1—C2—H2	120.1
O1^iii^—Nd1—O2^iii^	79.66 (7)	N2—C3—C4	120.2 (2)
O2^i^—Nd1—O2^iii^	118.92 (10)	N2—C3—H3	119.9
O2^ii^—Nd1—O2^iii^	72.37 (10)	C4—C3—H3	119.9
O2—Nd1—O2^iii^	145.02 (9)	N2^viii^—C4—C3	120.1 (2)
O3—Cl1—O3^iv^	109.83 (10)	N2^viii^—C4—H4	119.9
O3—Cl1—O3^iii^	108.76 (19)	C3—C4—H4	119.9

Symmetry codes: (i) *y*+1/4, *x*−1/4, −*z*+3/4; (ii) −*y*+3/4, −*x*+3/4, −*z*+3/4; (iii) −*x*+1, −*y*+1/2, *z*; (iv) −*y*+3/4, *x*−1/4, −*z*+1/4; (v) *y*+1/4, −*x*+3/4, −*z*+1/4; (vi) *y*+3/4, *x*−3/4, −*z*+1/4; (vii) −*x*+3/2, −*y*+1/2, −*z*+1/2; (viii) *x*, −*y*+1, −*z*+1/2.

### Hydrogen-bond geometry (Å, °)

**Table:**

*D*—H···*A*	*D*—H	H···*A*	*D*···*A*	*D*—H···*A*
C2—H2···O2^vii^	0.95	2.55	3.326 (3)	139.
C2—H2···O5	0.95	2.43	3.194 (7)	137.
C3—H3···O1	0.95	2.59	3.331 (3)	135.
C3—H3···O3	0.95	2.51	3.260 (3)	136.
C4—H4···O3^iv^	0.95	2.41	3.289 (3)	154.

Symmetry codes: (vii) −*x*+3/2, −*y*+1/2, −*z*+1/2; (iv) −*y*+3/4, *x*−1/4, −*z*+1/4.
